# Hip-hop dance participation and cognitive, gait, and muscle mass outcomes in community-dwelling older adults: a multi-site longitudinal study

**DOI:** 10.3389/fragi.2026.1862298

**Published:** 2026-07-10

**Authors:** Atsuko Miyazaki, Takashi Okuyama, Hayato Mori, Kazuhisa Sato, Sawako Ono, Atsushi Hiyama

**Affiliations:** 1 Research Center for Advanced Science and Technology, The University of Tokyo, Tokyo, Japan; 2 Super Reha, LLC., Tokyo, Japan; 3 WARAKU Inc., Tokyo, Japan; 4 yoga i.um Inc., Hyogo, Japan; 5 Graduate School of Social Data Science, Hitotsubashi University, Kunitachi, Japan

**Keywords:** body composition, cadence, cognitive function, gait, hip-hop dance, longitudinal study, older adults, sarcopenia

## Abstract

**Background:**

Dance-based interventions show promise for maintaining function in older adults, but long-term effects on body composition remain unclear. This study examined the effects of a hip-hop dance program on cognitive function, gait, and muscle mass over up to 3 years.

**Methods:**

This multi-site longitudinal study included 102 community-dwelling older adults (mean age 74.2 years, 93% female) from six sites in Japan. The dance group (*n* = 74) participated in weekly hip-hop dance sessions and competed in the FIDA GOLD CUP, a competition for older adults. Controls (*n* = 28) maintained usual activities. Outcomes included cognitive function (MMSE, MoCA visuospatial), gait parameters, physical function, and body composition (SMI, segmental lean mass, BMI). Linear mixed models examined group × time interactions, and ANCOVA with permutation tests compared change scores in participants with complete data (*n* = 59).

**Results:**

Significant group × time interactions favoring the dance group were observed for MMSE (*β* = 0.58 points/year, *p* = 0.007) and MoCA visuospatial (*β* = 0.27/year, *p* = 0.023). Gait improved through temporal parameters: left contact time decreased (*β* = −0.018 s/year, *p* = 0.005) and gait speed increased (*β* = 0.065 m/s/year, *p* = 0.031), while stride length remained unchanged (*p* = 0.243). Right hip abduction strength (*β* = 1.22 kg/year, *p* = 0.016) and right single-leg stance time (*β* = 10.3 s/year, *p* = 0.026) improved. However, the dance group showed greater decline in skeletal muscle mass index (*β* = −0.064 kg/m^2^/year, *p* = 0.020) and leg segmental lean mass (left: *β* = −0.108 kg/year, *p* < 0.001; right: *β* = −0.088 kg/year, *p* = 0.004), while BMI remained unchanged (*p* = 0.949). ANCOVA confirmed these patterns for cognition, gait, and body composition outcomes.

**Conclusion:**

Hip-hop dance participation was associated with better preservation of cognitive function and more favorable gait-related change, characterized by enhanced temporal control rather than increased stride length. Specifically, reduced contact time and faster cadence suggest rhythm-based motor learning as the underlying mechanism. However, dance participation alone was not sufficient to prevent age-related muscle loss, indicating the need for combined interventions incorporating resistance training and adequate protein intake.

## 1 Introduction

Population aging is one of the major demographic challenges facing health systems worldwide, with the number of adults aged 65 years and older projected to rise from 761 million in 2021 to 1.6 billion by 2050 (United Nations, 2023). In later life, cognitive decline, mobility limitation, and progressive loss of muscle mass can undermine functional independence and quality of life. Regular physical activity is among the most effective strategies for preserving these functions. However, maintaining long-term participation in conventional exercise programs remains challenging for many older adults (Picorelli et al., 2014). This issue is especially pressing in Japan, where population aging has advanced earlier and more rapidly than in many other countries, underscoring the need for physical activity programs that are both effective and sustainable in community settings.

Dance may be particularly suitable for older adults because it integrates movement with continuous cognitive engagement, sensorimotor synchronization, visuospatial adaptation, and social interaction within a cognitively enriched environment. Through repeated coupling of movement to rhythm, dance may support temporal motor control, while the demands of choreography, spatial coordination, and group performance may simultaneously engage cognitive processes relevant to later-life function (Hackney et al., 2024). Rhythmic movement interventions have been associated with improvements in both physical function, including gait speed and stride, and global cognition in older adults (Ma et al., 2023). In addition, structured dance has been shown to be at least as effective as other forms of physical activity for psychological and cognitive outcomes (Fong Yan et al., 2024). Previous reviews have also linked dance-based interventions in older adults to benefits in cognition, balance, mobility, and fall-related outcomes (Fernandez-Arguelles et al., 2015; Hewston et al., 2021; Hwang and Braun, 2015; Lu et al., 2024; Predovan et al., 2019). Psychological benefits have also been reported in the broader dance literature (Koch et al., 2019; Podolski et al., 2023).

Gait may be one outcome for which these features are especially relevant. Several intervention studies have reported improved walking performance after dance or dance-related training in older adults (Granacher et al., 2012; Pichierri et al., 2012). In parallel, music- and rhythm-based training has been shown to improve gait and fall-related outcomes in older adults (Trombetti et al., 2011), and rhythmic auditory cueing improves spatiotemporal gait parameters in healthy older adults (Ghai et al., 2018).

Most dance intervention studies in older adults have been relatively short, often lasting only a few months. As a result, it remains unclear whether these benefits are sustained over longer periods, especially for outcomes that may change more gradually. Another unresolved issue concerns gait. Although several studies have reported improved walking performance after dance training, few have examined how that improvement is achieved. Walking speed can increase through longer steps, faster stepping, or both, yet these components are rarely discussed separately. In our previous 4-week randomized controlled trial (Miyazaki et al., 2022), hip-hop dance improved gait speed and cognitive function compared with Nordic walking and control conditions. However, MMSE did not change, possibly because of the short intervention period and high baseline scores, and stride length did not increase despite faster walking. The mechanism underlying gait improvement therefore remained unclear. In the present study, we included additional measures of lower-extremity function and balance to examine which physical factors might explain any gait-related changes.

A further unresolved issue concerns body composition. Although dance involves repeated whole-body movement, it remains unclear whether such activity is sufficient to preserve muscle mass in older adults. In our previous trial (Miyazaki et al., 2022), amino acid supplementation was provided to all groups and muscle mass was maintained. Whether dance alone, without nutritional support, can attenuate age-related muscle loss therefore remains unclear. This question is clinically important because sarcopenia is common in later life and is associated with falls, disability, and mortality (Chen et al., 2020; Cruz-Jentoft and Sayer, 2019).

**FIGURE 1:**
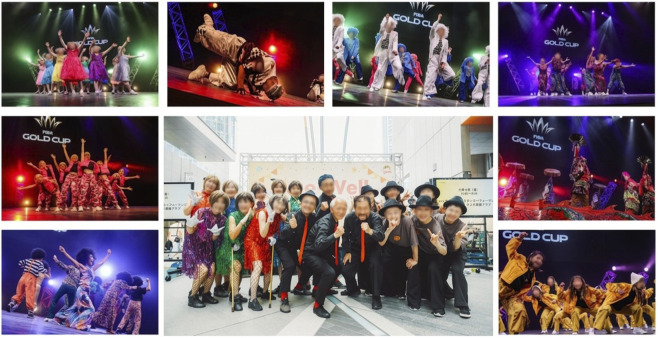
Participants performing in the FIDA GOLD CUP hip-hop dance competition in Tokyo. Photograph used with permission, courtesy of FIDA GOLD CUP.

The present study took advantage of a unique real-world context to address these questions. Participants were members of teams competing in the FIDA GOLD CUP, an annual nationwide hip-hop dance competition for older adults held in Tokyo (Figure 1). Unlike time-limited laboratory interventions, this program encourages sustained participation through team-based practice, choreography development, and the shared goal of public performance in competition. This setting provided an opportunity to examine the longer-term effects of dance participation under conditions of high ecological validity.

The aim of this study was to examine whether sustained participation in competitive hip-hop dance is associated with changes in cognitive function, gait parameters, body composition, psychological wellbeing, and the physical factors related to gait in community-dwelling older adults across three annual assessments. We hypothesized that continued dance participation would help preserve cognitive function and gait performance but might not be sufficient to prevent age-related muscle loss in the absence of nutritional support.

## 2 Methods

### 2.1 Study design and participants

This multi-site longitudinal study was conducted in Japan between January 2022 and October 2025. Participants were recruited from teams participating in the FIDA GOLD CUP, an annual hip-hop dance competition for older adults held in Tokyo, as well as from community-based control groups.

Seven sites were initially recruited from Osaka, Tokyo, Saitama, Aomori, Oita, Yamanashi, and Kanagawa prefectures. One site (Yamanashi, *n* = 8) was excluded because the team did not participate in the FIDA GOLD CUP after baseline assessment and had no follow-up data. The final analysis therefore included 102 participants from six sites.

Inclusion criteria were: (1) age 60 years or older, (2) ability to walk independently, (3) no diagnosis of dementia, and (4) ongoing participation in FIDA GOLD CUP-related activities for the dance group, or willingness to participate in repeated assessments for the control group. The dance group (*n* = 74) consisted of active competitors from six teams. The control group (*n* = 28) was recruited through municipal offices and social welfare councils in Osaka and Saitama and consisted of community-dwelling older adults who were not engaged in regular dance activities.

### 2.2 Hip-hop dance intervention

As of 2025, the FIDA GOLD CUP included more than 600 registered participants from 35 teams across 22 prefectures in Japan. The competition has expanded in recent years, with 13 teams participating in 2023, 16 teams in 2024, and 17 teams in 2025.

Each team developed its own choreography, selected its own music, and designed its own costumes for competition. Dance sessions were led by certified instructors and tailored for older adults, incorporating age-appropriate movements performed to hip-hop music. Teams practiced independently at their local sites throughout the year, although practice frequency and duration varied across teams and participants. The annual competition, held each autumn in Tokyo, served as a shared goal that encouraged sustained participation.

### 2.3 Outcome measures

Assessors were not informed of participants’ group assignment; at sites enrolling both dance and control participants, all individuals were assessed under the same conditions without distinction. To ensure measurement consistency, the same assessors were assigned to each site across time points wherever possible. Physical function assessments were conducted by two physical therapists per participant to ensure standardized administration and scoring.

#### 2.3.1 Cognitive function

Global cognitive function was assessed using the Mini-Mental State Examination (MMSE; Folstein et al., 1975). Higher-order cognitive function was assessed using the visuospatial/executive subscale of the Montreal Cognitive Assessment (MoCA; Nasreddine et al., 2005). Executive function was assessed using the Frontal Assessment Battery (FAB; Dubois et al., 2000).

#### 2.3.2 Gait parameters

Gait parameters were measured using the AYUMI EYE (Waseda Elderly Health Association Co., Ltd., Tokyo, Japan), a lightweight triaxial accelerometer (18.5 g; 62.4 × 30.9 × 11.8 mm) attached at the level of the third lumbar vertebra with a rubber belt. Participants walked along a 12-m walkway, with the central 10 m serving as the measurement distance and 1-m zones at each end allowing for acceleration and deceleration. Participants were instructed to walk as fast as possible without running. Fast walking was assessed because the ability to walk faster than usual may reflect gait reserve, which has been linked to cognitive status in older adults (Callisaya et al., 2017). Two trials were conducted, and mean values across trials were used for analysis. The following parameters were extracted: walking speed (m/s), stride length (m), gait cycle duration (s), and left and right contact time (s). Cadence (steps/min) was calculated as 120 divided by gait cycle duration.

#### 2.3.3 Physical function and lower extremity strength

Walking speed is determined by stride length and cadence. Prior research has shown that stride length declines with aging, whereas cadence may increase as a compensatory mechanism (Ko et al., 2010). To better understand gait-related changes, we additionally assessed lower-extremity function and balance.

##### 2.3.3.1 Heel-rise performance (heel-rise angle)

Participants stood barefoot facing a wall, with their hands lightly touching the wall at shoulder height for balance, feet hip-width apart, and knees fully extended. On command, they raised their heels to the maximum height and maintained the position for 5 s. Maximum heel height from the floor (h) was measured using a ruler. Foot length (L) was measured as the distance from the first metatarsophalangeal joint to the heel. Heel-rise angle was calculated as *θ* = arctan (h/L) and expressed in degrees. The heel-rise test is an established measure of plantar-flexor functional capacity (Bohannon, 2022; Hébert-Losier et al., 2017). This measure was included because plantar-flexor output is an important biomechanical factor related to stride length in older adults (Anderson and Madigan, 2014).

##### 2.3.3.2 Timed up and go test (TUG)

TUG was measured as the best of two trials. This test was included as an indicator of dynamic balance and functional mobility. Older adults with impaired balance or fear of falling may adopt shorter strides as a protective strategy (Makino et al., 2017).

##### 2.3.3.3 Single-leg standing time

Participants stood on one leg at a time on a flat surface while wearing shoes, with their eyes open. The maximum duration was 120 s. If balance was lost before 120 s, the elapsed time was recorded. Two trials were performed on each side, and the best time was used for analysis. Single-leg standing time was included as a simple indicator of balance and postural stability during single-limb support (Michikawa et al., 2009).

##### 2.3.3.4 Grip strength

Handgrip strength was measured bilaterally using a digital dynamometer (T.K.K.5401, Takei Scientific Instruments Co., Ltd., Niigata, Japan). Two maximum-effort trials were performed for each hand, and the highest value was used for analysis. Grip strength was included as a general indicator of overall muscle strength and sarcopenia rather than as a direct determinant of gait parameters (Bohannon, 2019; Chen et al., 2020).

##### 2.3.3.5 Knee extension strength

Isometric knee extension strength was measured bilaterally using a hand-held dynamometer (μTas F-1, Anima Corp., Tokyo, Japan). Two trials were performed for each leg, and the highest value was recorded. Knee extension strength was included as a lower-extremity strength measure relevant to gait performance (Goldberg and Neptune, 2007).

##### 2.3.3.6 Hip flexion strength

Isometric hip flexion strength was measured bilaterally using the same hand-held dynamometer (μTas F-1). Two trials were performed for each leg, and the highest value was recorded. Hip flexion strength was included because aging gait is characterized by a distal-to-proximal redistribution of joint torques and powers (DeVita and Hortobagyi, 2000).

##### 2.3.3.7 Hip abduction strength

Isometric hip abduction strength was measured bilaterally using the same hand-held dynamometer (μTas F-1). Two trials were performed for each leg, and the highest value was recorded. Hip abduction strength was included as an indicator of lateral stability during walking, particularly during single-limb support (Arvin et al., 2015).

#### 2.3.4 Body composition

Body composition was assessed using a bioelectrical impedance analyzer (InBody S10; Biospace Co., Ltd., Seoul, Korea). Skeletal muscle mass index (SMI) calculated as appendicular skeletal muscle mass divided by height squared (kg/m^2^), and segmental lean mass of the right and left legs (kg) were recorded. SMI was included because height-adjusted muscle mass is a core indicator used in the assessment of sarcopenia (Chen et al., 2020).

#### 2.3.5 Psychological measures

Depressive symptoms were assessed using the Japanese version of the 15-item Geriatric Depression Scale (GDS-15; Sheikh and Yesavage, 1986). The GDS-15 is a brief questionnaire designed for older adults and uses a yes/no response format. Total scores range from 0 to 15, with higher scores indicating more depressive symptoms (cutoff ≥5).

Life satisfaction was assessed using the Satisfaction with Life Scale (SWLS; Diener et al., 1985), a 5-item questionnaire rated on a 7-point scale that measures global cognitive judgments of life satisfaction.

Quality of life was assessed using the World Health Organization Quality of Life-BREF (WHOQOL-BREF; The WHOQOL Group, 1998), a 26-item self-administered questionnaire covering physical, psychological, social, and environmental domains, as well as overall quality of life. Each item was rated on a 5-point scale, and mean domain scores were calculated, with higher scores indicating better quality of life.

#### 2.3.6 Imitation ability

Imitation ability was assessed using a standardized hand gesture imitation task adapted from the meaningless gesture items of the Standard Performance Test for Apraxia (SPTA; Japan Society for Higher Brain Dysfunction Brain Function Test Committee, 1999). The examiner sat facing the participant and presented gestures to be imitated.

The task comprised three categories: one-handed tasks, two-handed tasks, and continuous one-handed movement tasks. One-handed tasks included Luria’s chin-hand test performed with each hand, a fox gesture forming a ring with fingers I, III, and IV with each hand, and formation of a ring with fingers I and V with left-to-right and right-to-left transfer (maximum observation time: 10 s each). Two-handed tasks included intertwining rings with fingers I and II of both hands, crossing the palms with fingers extended upward and intertwining both thumbs, and simultaneous rock-paper hand alternation tapping the desk three times at 2-s intervals (maximum observation time: 15 s each). Continuous one-handed movement tasks included the Luria flexion-ring and extension-fist sequence repeated three times (maximum observation time: 20 s).

Scoring was error-based: 0 points for perfect imitation, 1 point for self-corrected errors, and 2 points for incomplete imitation, yielding a total score ranging from 0 to 22, with higher scores indicating poorer performance.

### 2.4 Statistical analysis

Linear mixed models (LMMs) were used as the primary analysis to examine longitudinal change while accounting for repeated measures within individuals. Each outcome was modeled as a function of elapsed time from baseline (years), group (dance vs. control), and their interaction, with age, sex, and baseline value included as covariates. Random intercepts were included for participants. Because follow-up intervals varied across sites, elapsed time was calculated from each participant’s actual assessment dates rather than treated as fixed intervals. The group × time interaction was the primary parameter of interest and represented the annual difference in the rate of change between groups. For variables in which lower values indicated better performance (e.g., contact time, TUG), negative coefficients favored the dance group.

As a secondary analysis, analysis of covariance (ANCOVA) was used to compare change scores (final follow-up minus baseline) between groups, adjusting for age, sex, baseline value, and observation period. This analysis was restricted to participants with complete data at baseline and final follow-up (*n* = 59). To reduce sensitivity to non-normality and modest sample size, p values were obtained using permutation tests with 5,000 random permutations of group assignment. Partial eta-squared (*η* ^2^) was calculated as the effect size.

The Tokyo site completed baseline and mid-point assessments only and therefore contributed to the LMM analysis but not to the ANCOVA. All analyses were performed using R version 4.5.3 (R Core Team, 2025).

### 2.5 Ethical considerations

Before enrollment, written informed consent was obtained from all participants, and all study procedures were conducted in accordance with the Declaration of Helsinki. The study protocol was approved by the Research Ethics Committee of the University of Tokyo (approval numbers: 21-304 and 24-259).

## 3 Results

### 3.1 Participant characteristics

A total of 102 participants (mean age 74.2 ± 6.2 years, range 61–91; 93.1% female) were enrolled in the study and included in the longitudinal analyses. Participants were recruited from six dance teams participating in the FIDA GOLD CUP, an annual dance competition held in Tokyo: Osaka (*n* = 33), Tokyo (*n* = 20), Saitama (*n* = 16), Aomori (*n* = 13), Oita (*n* = 12), and Kanagawa (*n* = 8). Two teams (Osaka and Saitama) included both dance and control participants, while the remaining four teams enrolled dance participants only.

Of the 102 enrolled participants, 100 had complete baseline data and were included in baseline comparisons (dance, *n* = 73; control, *n* = 27). Baseline characteristics are presented in Table 1. Descriptive statistics for all outcome measures at each time point are presented in Table 2. The two groups were broadly similar at baseline, although some differences were observed in dance experience and selected physical measures. No participants exhibited slow gait at baseline (<1.0 m/s), and BMI was also similar between groups.

**TABLE 1:** Baseline characteristics of participants.

Variable	Dance (*n* = 74)	Control (*n* = 28)	*p*
Demographics			
Age, years	74.3 ± 6.1	74.2 ± 6.8	0.912
Female, *n* (%)	70 (96)	23 (85)	0.155
Education, years	13.1 ± 1.9	12.7 ± 2.0	0.436
Exercise history			
Regular exercise habit, *n* (%)	53 (73)	19 (70)	1.000
Dance experience, *n* (%)	51 (70)	7 (26)	**<0.001**
Health status			
Lower limb pain, *n* (%)	35 (48)	10 (37)	0.455
Lifestyle disease medication, *n* (%)	40 (55)	14 (52)	0.971
Diabetes, n (%)	3 (4)	1 (4)	1.000
Cognitive function			
MMSE	28.0 ± 1.8	28.3 ± 1.6	0.409
MoCA visuospatial	3.8 ± 1.1	3.8 ± 1.1	0.893
MoCA naming	2.9 ± 0.4	2.8 ± 0.6	0.450
MoCA attention	5.3 ± 0.8	5.2 ± 0.8	0.392
MoCA language	1.8 ± 0.7	1.9 ± 0.9	0.328
MoCA abstraction	1.4 ± 0.7	1.5 ± 0.7	0.527
MoCA memory	4.5 ± 0.8	4.5 ± 0.8	0.866
MoCA orientation	5.9 ± 0.3	6.0 ± 0.2	0.561
MoCA total	25.7 ± 2.6	25.8 ± 2.2	0.889
FAB similarities	2.0 ± 0.9	1.6 ± 0.9	0.057
FAB lexical fluency	2.7 ± 0.5	2.7 ± 0.5	0.624
FAB motor series	2.6 ± 0.7	2.2 ± 1.1	**0.023**
FAB conflicting instructions	2.8 ± 0.5	2.8 ± 0.4	0.816
FAB go-no-go	2.3 ± 0.9	2.3 ± 0.7	0.866
FAB prehension	3.0 ± 0.0	3.0 ± 0.0	​
FAB total	15.3 ± 2.1	14.6 ± 2.0	0.143
Gait			
Gait speed, m/s	1.9 ± 0.4	1.8 ± 0.3	0.292
Stride length, cm	74.9 ± 11.4	73.7 ± 9.4	0.624
Cadence, steps/min	153.9 ± 21.3	149.1 ± 17.1	0.298
Contact time L, s	0.4 ± 0.1	0.4 ± 0.0	0.928
Contact time R, s	0.4 ± 0.1	0.4 ± 0.0	0.097
Balance and strength			
Heel-rise angle	39.5 ± 3.3	39.0 ± 4.4	0.596
TUG, s	6.0 ± 1.2	6.6 ± 1.3	0.051
One-leg stance R, s	41.7 ± 44.6	62.9 ± 47.9	**0.042**
One-leg stance L, s	32.4 ± 39.2	47.1 ± 44.6	0.113
Hip abduction R, kg	20.5 ± 5.6	20.5 ± 5.3	0.970
Hip abduction L, kg	21.6 ± 13.6	19.7 ± 4.9	0.476
Hip flexion R, kg	14.8 ± 4.8	17.3 ± 25.7	0.425
Hip flexion L, kg	13.9 ± 4.1	10.9 ± 5.5	**0.005**
Knee extension R, kg	19.1 ± 5.5	19.5 ± 5.4	0.744
Knee extension L, kg	18.9 ± 5.8	20.3 ± 6.2	0.284
Grip strength R, kg	20.1 ± 3.8	21.7 ± 5.3	0.102
Grip strength L, kg	20.0 ± 4.7	21.4 ± 5.7	0.199
Body composition			
BMI, kg/m^2^	22.6 ± 3.2	22.4 ± 2.7	0.788
SMI, kg/m^2^	6.1 ± 0.6	6.0 ± 0.7	0.543
Segmental lean RL, kg	5.5 ± 0.8	5.3 ± 1.1	0.555
Segmental lean LL, kg	5.5 ± 0.8	5.3 ± 1.1	0.378
Psychological			
WHOQOL physical	27.1 ± 3.9	27.9 ± 4.0	0.343
WHOQOL psychological	23.1 ± 4.0	21.7 ± 4.3	0.129
WHOQOL social	11.5 ± 2.2	11.6 ± 2.0	0.966
WHOQOL environment	30.6 ± 4.2	29.2 ± 8.1	0.272
WHOQOL overall	7.6 ± 1.5	7.2 ± 1.3	0.243
WHOQOL total	99.4 ± 12.9	97.6 ± 16.4	0.554
GDS	1.5 ± 2.0	2.3 ± 3.1	0.172
SWLS	26.2 ± 5.6	26.3 ± 6.7	0.972
Other			
Imitation score	1.7 ± 2.3	2.1 ± 2.7	0.527

Values are mean ± SD for continuous variables or *n* (%) for categorical variables. *p* values were obtained using independent *t* tests or chi-square tests, as appropriate. Bold indicates *p* < 0.05. Baseline comparisons were based on participants with complete baseline data (dance, *n* = 73; control, *n* = 27). Two participants without complete baseline assessment were included only in the longitudinal analyses and were not included in baseline comparisons (total *n* = 102).

**TABLE 2:** Descriptive statistics of outcome measures at each time point.

Variable	Group	T1	T2	T3
Cognitive function				
MMSE	Dance	28.0 ± 1.8	27.8 ± 1.9	28.1 ± 1.6
	Control	28.3 ± 1.6	28.0 ± 1.8	27.2 ± 2.2
MoCA visuospatial	Dance	3.8 ± 1.1	4.1 ± 0.9	4.3 ± 0.9
	Control	3.8 ± 1.1	3.5 ± 1.0	3.7 ± 1.0
MoCA naming	Dance	2.9 ± 0.4	2.9 ± 0.3	2.9 ± 0.2
	Control	2.8 ± 0.6	2.8 ± 0.4	2.9 ± 0.3
MoCA attention	Dance	5.3 ± 0.8	5.4 ± 0.9	5.5 ± 0.8
	Control	5.2 ± 0.8	5.4 ± 0.9	5.4 ± 0.9
MoCA language	Dance	1.8 ± 0.7	1.8 ± 0.7	1.9 ± 0.7
	Control	1.9 ± 0.9	1.9 ± 0.7	2.2 ± 0.7
MoCA abstraction	Dance	1.4 ± 0.7	1.6 ± 0.5	1.8 ± 0.4
	Control	1.5 ± 0.7	1.5 ± 0.6	1.6 ± 0.6
MoCA memory	Dance	4.5 ± 0.8	4.5 ± 1.1	4.5 ± 0.9
	Control	4.5 ± 0.8	4.8 ± 0.4	4.7 ± 0.6
MoCA orientation	Dance	5.9 ± 0.3	5.8 ± 0.4	5.8 ± 0.5
	Control	6.0 ± 0.2	5.9 ± 0.3	6.0 ± 0.5
MoCA total	Dance	25.7 ± 2.6	26.2 ± 2.6	26.0 ± 5.2
	Control	25.8 ± 2.2	25.8 ± 2.2	26.5 ± 2.5
FAB similarities	Dance	2.0 ± 0.9	2.0 ± 0.8	2.2 ± 0.6
	Control	1.6 ± 0.9	1.8 ± 0.9	2.0 ± 0.8
FAB lexical fluency	Dance	2.7 ± 0.5	2.7 ± 0.5	2.8 ± 0.4
	Control	2.7 ± 0.5	2.8 ± 0.4	2.8 ± 0.5
FAB motor series	Dance	2.6 ± 0.7	2.8 ± 0.4	2.9 ± 0.4
	Control	2.2 ± 1.1	2.8 ± 0.5	2.7 ± 0.6
FAB conflicting instructions	Dance	2.8 ± 0.5	2.7 ± 0.7	3.0 ± 0.2
	Control	2.8 ± 0.4	2.8 ± 0.4	2.8 ± 0.5
FAB go-no-go	Dance	2.3 ± 0.9	2.6 ± 0.7	2.6 ± 0.7
	Control	2.3 ± 0.7	2.2 ± 0.8	2.6 ± 0.6
FAB prehension	Dance	3.0 ± 0.0	3.0 ± 0.4	3.0 ± 0.0
	Control	3.0 ± 0.0	3.0 ± 0.0	3.0 ± 0.0
FAB total	Dance	15.3 ± 2.1	15.7 ± 1.9	15.5 ± 4.0
​	Control	14.6 ± 2.0	15.4 ± 1.8	16.0 ± 1.5
Gait				
Gait speed, m/s	Dance	1.9 ± 0.4	1.9 ± 0.4	2.0 ± 0.4
	Control	1.8 ± 0.3	1.8 ± 0.3	1.7 ± 0.3
Stride length, cm	Dance	74.9 ± 11.4	74.5 ± 10.4	74.4 ± 9.7
	Control	73.7 ± 9.4	73.3 ± 8.5	70.6 ± 9.7
Cadence, steps/min	Dance	153.9 ± 21.3	156.2 ± 17.0	160.5 ± 27.7
	Control	149.1 ± 17.1	147.7 ± 18.1	146.2 ± 15.1
Contact time L, s	Dance	0.4 ± 0.1	0.4 ± 0.0	0.4 ± 0.1
	Control	0.4 ± 0.0	0.4 ± 0.1	0.4 ± 0.0
Contact time R, s	Dance	0.4 ± 0.1	0.4 ± 0.0	0.4 ± 0.1
​	Control	0.4 ± 0.0	0.4 ± 0.1	0.4 ± 0.0
Balance and strength				
Heel angle, °	Dance	39.5 ± 3.3	39.1 ± 3.6	38.9 ± 3.0
	Control	39.0 ± 4.4	39.1 ± 2.8	39.3 ± 3.2
TUG, s	Dance	6.0 ± 1.2	5.8 ± 1.1	6.0 ± 1.1
	Control	6.6 ± 1.3	6.4 ± 1.3	6.2 ± 1.6
One-leg stance R, s	Dance	41.7 ± 44.6	45.1 ± 43.0	44.9 ± 36.6
	Control	62.9 ± 47.9	56.2 ± 46.4	44.5 ± 44.4
One-leg stance L, s	Dance	32.4 ± 39.2	39.8 ± 40.6	30.9 ± 37.6
	Control	47.1 ± 44.6	46.4 ± 48.4	53.6 ± 50.2
Hip abduction R, kg	Dance	20.5 ± 5.6	21.1 ± 5.3	23.0 ± 4.9
	Control	20.5 ± 5.3	19.8 ± 5.5	20.0 ± 6.1
Hip abduction L, kg	Dance	21.6 ± 13.6	23.3 ± 4.9	22.0 ± 5.1
	Control	19.7 ± 4.9	20.2 ± 5.1	20.0 ± 5.8
Hip flexion R, kg	Dance	14.8 ± 4.8	14.8 ± 4.0	18.4 ± 17.4
	Control	17.3 ± 25.7	15.4 ± 4.5	14.9 ± 4.3
Hip flexion L, kg	Dance	13.9 ± 4.1	13.9 ± 4.4	15.0 ± 4.9
	Control	10.9 ± 5.5	14.6 ± 4.5	13.7 ± 3.8
Knee extension R, kg	Dance	19.1 ± 5.5	19.4 ± 4.8	22.3 ± 6.6
	Control	19.5 ± 5.4	18.3 ± 5.9	20.7 ± 6.1
Knee extension L, kg	Dance	18.9 ± 5.8	20.8 ± 7.5	22.3 ± 6.7
	Control	20.3 ± 6.2	20.1 ± 6.5	20.8 ± 6.6
Grip strength R, kg	Dance	20.1 ± 3.8	21.3 ± 4.3	22.4 ± 4.6
	Control	21.7 ± 5.3	20.5 ± 6.9	21.6 ± 4.8
Grip strength L, kg	Dance	20.0 ± 4.7	20.8 ± 4.0	21.2 ± 4.0
​	Control	21.4 ± 5.7	21.2 ± 6.2	21.7 ± 4.6
Body composition				
BMI, kg/m^2^	Dance	22.6 ± 3.2	22.6 ± 3.3	23.2 ± 2.7
	Control	22.4 ± 2.7	22.2 ± 2.6	22.1 ± 2.6
SMI, kg/m^2^	Dance	6.1 ± 0.6	6.0 ± 0.6	6.2 ± 0.6
	Control	6.0 ± 0.7	6.1 ± 0.8	6.0 ± 0.8
Segmental lean RL, kg	Dance	5.5 ± 0.8	5.3 ± 0.8	5.5 ± 0.9
	Control	5.3 ± 1.1	5.4 ± 1.2	5.3 ± 1.1
Segmental lean LL, kg	Dance	5.5 ± 0.8	5.4 ± 0.9	5.5 ± 0.9
​	Control	5.3 ± 1.1	5.4 ± 1.2	5.3 ± 1.1
Psychological				
WHOQOL physical	Dance	27.1 ± 3.9	27.7 ± 3.7	27.7 ± 3.9
	Control	27.9 ± 4.0	27.4 ± 4.5	27.3 ± 3.7
WHOQOL psychological	Dance	23.1 ± 4.0	23.5 ± 3.4	24.1 ± 3.1
	Control	21.7 ± 4.3	22.2 ± 4.6	21.9 ± 3.8
WHOQOL social	Dance	11.5 ± 2.2	12.1 ± 1.8	12.3 ± 1.5
	Control	11.6 ± 2.0	11.2 ± 1.7	11.3 ± 2.0
WHOQOL environment	Dance	30.6 ± 4.2	31.2 ± 4.5	32.0 ± 5.1
	Control	29.2 ± 8.1	30.5 ± 5.2	30.4 ± 4.8
WHOQOL overall	Dance	7.6 ± 1.5	7.4 ± 1.4	7.7 ± 1.4
	Control	7.2 ± 1.3	7.6 ± 1.3	7.2 ± 1.1
WHOQOL total	Dance	99.4 ± 12.9	101.9 ± 12.6	100.9 ± 21.8
	Control	97.6 ± 16.4	99.0 ± 16.1	98.2 ± 13.2
GDS	Dance	1.5 ± 2.0	1.4 ± 2.3	1.0 ± 2.0
	Control	2.3 ± 3.1	1.7 ± 2.9	1.8 ± 2.8
SWLS	Dance	26.2 ± 5.6	27.1 ± 5.2	28.9 ± 11.0
​	Control	26.3 ± 6.7	25.2 ± 6.6	24.8 ± 6.4
Other				
Imitation score	Dance	1.7 ± 2.3	1.1 ± 1.8	1.2 ± 2.0
	Control	2.1 ± 2.7	1.6 ± 2.3	1.6 ± 2.0

Values are mean ± SD. T1 = baseline, T2 = mid (approximately 1 year), T3 = final (approximately 2 years). Sample sizes varied by variable due to missing data; Dance *n* = 73/60-62/34-35, Control *n* = 27/25-26/25-26 at T1/T2/T3.

Dance training characteristics. Prior to baseline assessment, duration of dance team participation varied: less than 1 year (*n* = 33, 45.2%), 1–2 years (*n* = 12, 16.4%), and 2–3 years (*n* = 28, 38.4%). At baseline, participants reported attending team practice sessions a median of 1.0 time per week (IQR: 0.7–2.0) for approximately 30 min per session, with additional home practice of 1.0 time per week (IQR: 1.0–2.0) for 15 min. Total estimated dance practice time was approximately 75 min per week.

Regarding sarcopenia-related indices, skeletal muscle mass index (SMI) was comparable between groups (dance: 6.1 ± 0.6 kg/m^2^; control: 6.0 ± 0.7 kg/m^2^; *p* = 0.543). Grip strength was 19.9 ± 4.5 kg in the dance group and 20.9 ± 6.7 kg in the control group, with approximately one-third of participants meeting the AWGS 2019 criteria for low grip strength. Overall, 12.7% of participants met the criteria for sarcopenia, with no significant difference between groups.

Baseline characteristics are presented in Table 1. Dance training characteristics were assessed by questionnaire in the dance group only.

### 3.2 Participant flow

Assessments were conducted at three time points: baseline (T1), mid-point (T2), and final follow-up (T3). Due to the nature of the annual competition schedule, measurement intervals varied across teams. The interval from T1 to T2 ranged from 0.93 to 1.38 years (mean 1.07 ± 0.15 years), and from T1 to T3 ranged from 1.92 to 2.91 years (mean 2.07 ± 0.26 years).

Of the 102 participants enrolled at baseline (T1), 88 (86.3%) completed the mid-point assessment at approximately 1 year (T2). Fourteen participants were lost to follow-up: 12 from the dance group (Tokyo: 4, Aomori: 3, Saitama: 2, Osaka: 2, Oita: 1) and two from the control group (Saitama: 1, Osaka: 1).

At the final follow-up at approximately 2 years (T3), 61 participants (59.8%) were assessed. Twenty-seven participants were not assessed at T3, all from the dance group. Of these, 16 were from the Tokyo team, which continued dancing but discontinued competition participation after the first year; as study eligibility required ongoing competition participation, this team was not assessed at T3. The remaining 11 participants were lost to follow-up from other teams (Aomori: 5, Saitama: 2, Osaka: 2, Kanagawa: 2). No control group participants were lost between T2 and T3.

**FIGURE 2:**
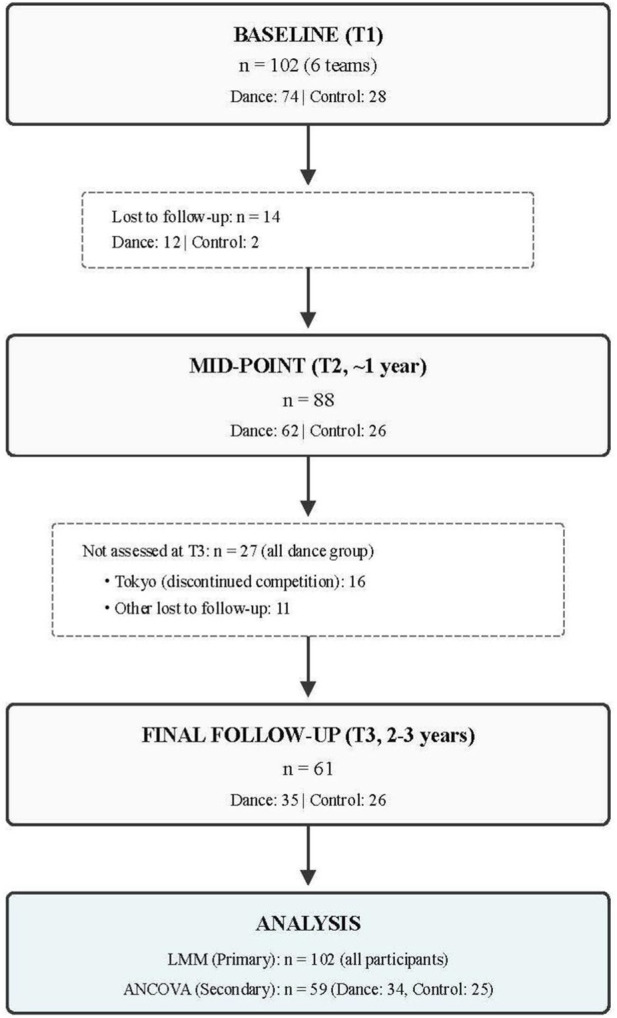
Participant flow diagram.

The mean observation period from baseline to final follow-up was 2.07 years (range: 1.92–2.91 years). Participant flow is presented in Figure 2.

### 3.3 Primary outcomes: linear mixed model analysis

**FIGURE 3:**
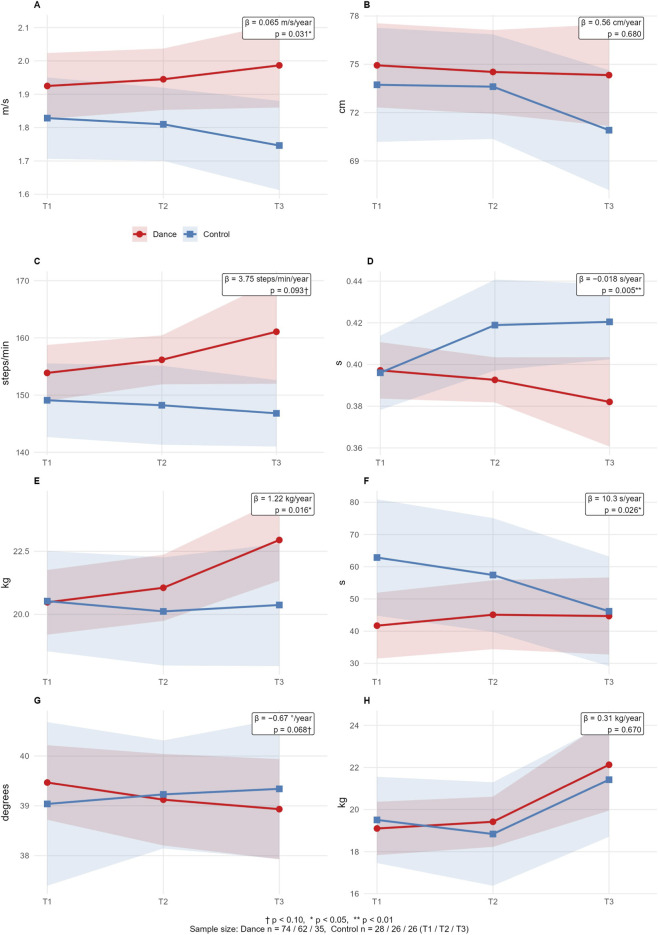
Gait and physical function outcomes across three assessment points by group. Red lines represent the dance group; blue lines represent the control group. Shaded areas indicate 95% confidence intervals. *β* = group × time interaction coefficient from linear mixed models; *†* < 0.10, *p < 0.05, **p < 0.01. **(A)** Gait speed. **(B)** Stride length. **(C)** Cadence. **(D)** Contact time (L). **(E)** Hip abduction (R). **(F)** One-leg stance (R). **(G)** Heel-rise angle. **(H)** Knee extension (R).

Linear mixed models examining group × time interactions across 45 outcomes revealed significant effects (*p* < 0.05) in 9 variables, as summarized in Table 3 and visualized in Figure 3.

**TABLE 3:** Linear mixed model results for group × time interaction.

Outcome	*N*	*β*	95% CI	*p*	*β*_std
Cognitive function					
MMSE	102	0.584	(0.168, 0.999)	0.007**	0.32
MoCA visuospatial	102	0.266	(0.039, 0.493)	0.023*	0.26
MoCA naming	102	−0.0203	(−0.1160, 0.0754)	0.678	−0.05
MoCA attention	102	−0.0412	(−0.2507, 0.1684)	0.701	−0.05
MoCA language	102	−0.0509	(−0.2476, 0.1458)	0.612	−0.07
MoCA abstraction	102	0.154	(−0.008, 0.315)	0.063^†^	0.26
MoCA memory	102	−0.127	(−0.336, 0.082)	0.236	−0.15
MoCA orientation	102	−0.0720	(−0.1883, 0.0443)	0.226	−0.19
MoCA total	102	0.0824	(−0.3871, 0.5519)	0.731	0.03
FAB similarities	102	−0.139	(−0.347, 0.069)	0.191	−0.16
FAB lexical fluency	102	−0.0257	(−0.1475, 0.0960)	0.679	−0.05
FAB motor series	102	−0.153	(−0.320, 0.015)	0.076^†^	−0.23
FAB conflicting	102	0.0272	(−0.1063, 0.1607)	0.690	0.05
FAB go-no-go	102	0.0145	(−0.1624, 0.1914)	0.873	0.02
FAB prehension	102	−0.0018	(−0.0645, 0.0608)	0.955	−0.01
FAB total	102	−0.277	(−0.649, 0.096)	0.147	−0.14
Gait parameters					
Gait speed (m/s)	100	0.0647	(0.0064, 0.1230)	0.031*	0.17
Stride length (cm)	100	1.03	(−0.70, 2.76)	0.243	0.10
Cadence (steps/min)	100	3.75	(−0.59, 8.09)	0.092^†^	0.18
Contact time L (s)	100	−0.0175	(−0.0295, −0.0056)	0.005**	−0.32
Contact time R (s)	100	0.0009	(−0.0112, 0.0129)	0.886	0.02
Balance and strength					
Heel-rise angle (°)	100	−0.671	(−1.386, 0.044)	0.068^†^	−0.20
Timed up and go (s)	100	0.137	(−0.021, 0.294)	0.090^†^	0.11
One-leg stance R (s)	100	10.34	(1.34, 19.33)	0.026*	0.24
One-leg stance L (s)	100	−1.47	(−9.60, 6.67)	0.724	−0.03
Hip abduction R (kg)	100	1.22	(0.24, 2.20)	0.016*	0.22
Hip abduction L (kg)	100	−0.443	(−2.845, 1.953)	0.717	−0.05
Hip flexion R (kg)	100	2.69	(−0.34, 5.73)	0.083^†^	0.24
Hip flexion L (kg)	100	−0.996	(−1.988, −0.004)	0.051^†^	−0.22
Knee extension R (kg)	100	0.628	(−0.560, 1.816)	0.301	0.11
Knee extension L (kg)	99	0.944	(−0.406, 2.293)	0.172	0.14
Grip strength R (kg)	102	0.763	(−0.025, 1.551)	0.059^†^	0.15
Grip strength L (kg)	101	0.111	(−0.540, 0.762)	0.739	0.02
Body composition					
BMI (kg/m^2^)	100	0.0065	(−0.1906, 0.2036)	0.949	0.00
SMI (kg/m^2^)	100	−0.0638	(−0.1173, −0.0104)	0.020*	−0.10
Segmental lean RL (kg)	100	−0.0879	(−0.1467, −0.0291)	0.004**	−0.10
Segmental lean LL (kg)	100	−0.108	(−0.1650, −0.0510)	<0.001**	−0.11
Psychological wellbeing					
WHOQOL physical	102	0.524	(−0.202, 1.250)	0.159	0.13
WHOQOL psychological	102	0.108	(−0.635, 0.851)	0.776	0.03
WHOQOL social	102	0.367	(−0.094, 0.828)	0.120	0.19
WHOQOL environment	102	−0.145	(−1.264, 0.975)	0.800	−0.03
WHOQOL overall	102	−0.0024	(−0.3183, 0.3135)	0.988	−0.00
WHOQOL total	102	0.908	(−1.608, 3.424)	0.480	0.07
SWLS	100	1.04	(−0.64, 2.71)	0.227	0.15
GDS	102	0.286	(−0.135, 0.707)	0.185	0.13
Other					
Imitation score	102	0.0087	(−0.5631, 0.5806)	0.976	0.00

*β* represents the annual difference in change between dance and control groups. Positive values indicate greater improvement (or less decline) in the dance group. *β*_std = standardized beta *(β/SD)*. **p < 0.01, *p < 0.05, ^†^ *p* < 0.10. Bold indicates statistical significance (*p* < 0.05).

#### 3.3.1 Cognitive function

MMSE demonstrated a significant group × time interaction (*β* = 0.58 points/year, 95% CI [0.17, 1.00], *p* = 0.007, *β*_std = 0.32). MoCA visuospatial subscale also showed a significant interaction (*β* = 0.27, 95% CI [0.04, 0.49], *p* = 0.023, *β*_std = 0.26). MoCA abstraction showed a trend (*β* = 0.15, *p* = 0.063). Other MoCA subscales (naming, attention, language, memory, orientation) and MoCA total did not reach significance (all *p* > 0.10). FAB motor showed a trend (*β* = −0.15, *p* = 0.076). Other FAB measures did not reach significance (all *p* > 0.10).

#### 3.3.2 Gait parameters

Left contact time showed a significant group × time interaction (*β* = −0.018 s/year, 95% CI [−0.029, −0.006], *p* = 0.005, *β*_std = −0.32; Figure 3D). Gait speed also showed a significant interaction (*β* = 0.065 m/s/year, 95% CI [0.006, 0.123], *p* = 0.031, *β*_std = 0.17; Figure 3A). Cadence showed a trend (*β* = 3.75 steps/min/year, *p* = 0.092; Figure 3C). Right contact time did not reach significance (*p* = 0.886). Stride length did not reach significance (*p* = 0.243; Figure 3B).

#### 3.3.3 Balance and strength

Right hip abduction strength showed a significant interaction (*β* = 1.22 kg/year, 95% CI [0.24, 2.20], *p* = 0.016, *β*_std = 0.22; Figure 3E). Right single-leg stance time also showed a significant interaction (*β* = 10.3 s/year, 95% CI [1.3, 19.3], *p* = 0.026, *β*_std = 0.24; Figure 3F). Left hip flexion showed a trend favoring the control group (*β* = −1.00, *p* = 0.051). Right hip flexion showed a trend (*β* = 2.69, *p* = 0.083). Right grip strength showed a trend (*β* = 0.76 kg/year, *p* = 0.059). TUG showed a trend favoring the control group (*β* = 0.14, *p* = 0.091). Heel angle showed a trend favoring the control group (*β* = −0.67°/year, *p* = 0.068; Figure 3G). Left one-leg stance, left hip abduction, knee extension, and left grip strength did not reach significance (all *p* > 0.10), although knee extension is shown in Figure 3H.

#### 3.3.4 Body composition

Left leg segmental lean mass showed a significant interaction (*β* = −0.108 kg/year, 95% CI [−0.165, −0.051], *p* < 0.001, *β*_std = −0.12), with the dance group showing greater decline. Right leg segmental lean mass showed a similar pattern (*β* = −0.088, 95% CI [−0.147, −0.029], *p* = 0.004, *β*_std = −0.10). SMI also showed greater decline in the dance group (*β* = −0.064 kg/m^2^/year, 95% CI [−0.117, −0.010], *p* = 0.020, *β*_std = −0.10). BMI did not differ significantly (*p* = 0.949).

#### 3.3.5 Psychological wellbeing

No WHOQOL-BREF domains or SWLS showed significant group × time interactions (all *p* > 0.10). The social relationships domain showed *β* = 0.37 (*p* = 0.120). GDS did not show a significant interaction (*β* = 0.29, *p* = 0.185, *β*_std = 0.13), though both groups maintained low depressive symptom levels throughout the study period. SWLS did not reach significance (*β* = 1.04, *p* = 0.227).

#### 3.3.6 Other measures

Imitation score did not show a significant interaction (*p* = 0.976).

### 3.4 Secondary outcomes: ANCOVA analysis

ANCOVA with permutation test examining change scores from baseline to final follow-up (*n* = 59; dance: *n* = 34, control: *n* = 25) revealed significant group differences in nine variables (Table 4).

**TABLE 4:** ANCOVA results for change from baseline to final follow-up.

Outcome	Dance *Δ* (SD)	Control *Δ* (SD)	*F*	ANCOVA Permutation *p*	*η* ^2^
Cognitive function					
MMSE	0.06 (1.69)	−1.08 (1.87)	7.55	0.022	0.125
MoCA visuospatial	0.44 (0.93)	−0.08 (0.95)	6.80	0.030	0.114
MoCA naming	0.00 (0.25)	0.08 (0.70)	1.20	0.641	0.022
MoCA attention	0.18 (0.94)	0.24 (0.93)	0.09	0.883	0.002
MoCA language	0.18 (0.83)	0.24 (0.93)	0.13	0.776	0.002
MoCA abstraction	0.44 (0.79)	0.08 (0.95)	11.11	0.125	0.173
MoCA memory	−0.15 (0.86)	0.20 (1.12)	2.95	0.183	0.053
MoCA orientation	−0.15 (0.61)	0.00 (0.58)	1.55	0.290	0.028
MoCA total	0.94 (2.30)	0.76 (2.18)	0.12	0.760	0.002
FAB similarities	0.26 (1.08)	0.46 (0.83)	1.07	0.449	0.020
FAB lexical fluency	0.03 (0.52)	0.08 (0.50)	0.24	0.691	0.005
FAB motor series	0.26 (0.79)	0.46 (1.10)	2.27	0.412	0.042
FAB conflicting	0.06 (0.34)	0.00 (0.51)	0.44	0.536	0.008
FAB go-no-go	0.15 (0.66)	0.25 (0.68)	0.54	0.676	0.010
FAB prehension	0.00 (0.00)	0.00 (0.00)	—	—	—
FAB total	0.76 (1.76)	1.25 (1.67)	2.71	0.283	0.050
Gait parameters					
Gait speed (m/s)	0.03 (0.26)	−0.09 (0.29)	3.07	0.018	0.055
Stride length (cm)	−1.06 (9.51)	−2.92 (7.90)	0.82	0.430	0.015
Cadence (steps/min)	4.55 (26.51)	−2.69 (17.95)	1.61	0.045	0.030
Contact time L (s)	−0.01 (0.07)	0.02 (0.04)	5.22	0.033	0.090
Contact time R (s)	−0.00 (0.06)	−0.01 (0.05)	0.22	0.685	0.004
Heel-rise angle (°)	−0.63 (2.23)	0.58 (4.93)	2.59	0.239	0.052
Balance and strength					
Timed up and go (s)	0.04 (0.67)	−0.28 (0.97)	2.18	0.144	0.040
One-leg stance R (s)	−2.23 (46.18)	−19.23 (38.07)	3.37	0.144	0.060
One-leg stance L (s)	−2.01 (30.51)	4.50 (31.06)	0.67	0.418	0.012
Hip abduction R (kg)	2.82 (4.63)	−0.36 (4.62)	8.75	0.013	0.142
Hip abduction L (kg)	−1.14 (20.28)	0.74 (4.87)	1.72	0.907	0.031
Hip flexion R (kg)	4.66 (17.64)	−2.99 (25.97)	4.35	0.296	0.076
Hip flexion L (kg)	1.74 (3.68)	2.78 (4.94)	1.19	0.368	0.022
Knee extension R (kg)	3.44 (7.31)	1.10 (3.88)	2.53	0.146	0.046
Knee extension L (kg)	3.26 (5.73)	0.62 (5.97)	3.55	0.092	0.063
Grip strength R (kg)	1.74 (3.05)	0.34 (3.21)	3.80	0.092	0.067
Grip strength L (kg)	1.36 (2.66)	0.88 (2.37)	0.67	0.478	0.013
Body composition					
BMI (kg/m^2^)	0.07 (1.18)	−0.09 (0.95)	0.32	0.572	0.006
SMI (kg/m^2^)	−0.07 (0.24)	0.02 (0.28)	1.92	0.192	0.035
Segmental lean RL (kg)	−0.11 (0.26)	0.06 (0.27)	5.88	0.025	0.100
Segmental lean LL (kg)	−0.12 (0.24)	0.08 (0.30)	8.74	0.006	0.142
Psychological wellbeing					
WHOQOL physical	0.24 (3.94)	−0.76 (3.03)	1.37	0.292	0.025
WHOQOL psychological	0.79 (3.85)	0.28 (4.06)	0.41	0.625	0.008
WHOQOL social	0.59 (1.97)	−0.28 (2.25)	3.86	0.046	0.068
WHOQOL environment	1.26 (5.52)	1.24 (7.73)	0.00	0.984	0.000
WHOQOL overall	−0.03 (1.27)	−0.08 (1.35)	0.04	0.898	0.001
WHOQOL total	2.85 (14.23)	0.40 (12.96)	0.69	0.495	0.013
GDS	0.06 (1.77)	−0.52 (2.55)	1.18	0.328	0.022
SWLS	1.24 (12.29)	−1.00 (4.30)	0.85	0.519	0.016
Other					
Imitation score	−0.03 (2.61)	−0.24 (2.71)	0.15	0.767	0.003

*Δ* = mean change (SD) from baseline. *F* = *F*-statistic*. η* ^2^ = partial eta-squared. Covariates: age, sex, baseline value, and observation period. *p*-values from permutation test (5,000 permutations).

#### 3.4.1 Cognitive function

MMSE showed a significant group difference (dance *Δ* = +0.06, control *Δ* = −1.08; *F* = 7.55, *p* = 0.022, *η* ^2^ = 0.125). MoCA visuospatial subscale also showed a significant group difference (dance *Δ* = +0.44, control *Δ* = −0.08; *F* = 6.80, *p* = 0.030, *η* ^2^ = 0.114). Other cognitive measures did not show significant group differences (all *p* > 0.10).

#### 3.4.2 Gait parameters

Gait speed showed a significant group difference (dance *Δ* = +0.03 ± 0.26 m/s, control *Δ* = −0.09 ± 0.29 m/s; *F* = 3.07, *p* = 0.018, *η* ^2^ = 0.055). Left contact time showed a significant difference (dance *Δ* = −0.01 ± 0.07 s, control *Δ* = +0.02 ± 0.04 s; *F* = 5.22, *p* = 0.033, *η* ^2^ = 0.090). Cadence showed a significant difference (dance *Δ* = +4.55 ± 26.51 steps/min, control *Δ* = −2.69 ± 17.95 steps/min; *F* = 1.61, *p* = 0.045, *η* ^2^ = 0.030). Stride length and right contact time did not reach significance (*p* > 0.05).

#### 3.4.3 Balance and strength

Right hip abduction showed a significant group difference (dance *Δ* = +2.82 kg, control *Δ* = −0.36 kg; *F* = 8.75, *p* = 0.013, *η* ^2^ = 0.142). Left knee extension (*p* = 0.092) and right grip strength (*p* = 0.092) showed trends. Other balance and strength measures did not reach significance.

#### 3.4.4 Body composition

Both segmental lean mass measures showed significant group differences, with the dance group exhibiting greater decline than controls. Left leg segmental lean mass (dance *Δ* = −0.12 kg, control *Δ* = +0.08 kg; *F* = 8.74, *p* = 0.006, *η* ^2^ = 0.142) and right leg segmental lean mass (dance *Δ* = −0.11 kg, control *Δ* = +0.06 kg; *F* = 5.88, *p* = 0.025, *η* ^2^ = 0.100) both reached significance.

#### 3.4.5 Psychological wellbeing

The social relationships domain of WHOQOL-BREF showed a significant group difference (dance *Δ* = +0.59 ± 1.97, control *Δ* = −0.28 ± 2.25; *F* = 3.86, *p* = 0.046, *η* ^2^ = 0.068). Other WHOQOL domains (physical, psychological, environment, overall, total) did not reach significance (all *p* > 0.10). GDS did not show a significant group difference (dance *Δ* = +0.06 ± 1.77, control *Δ* = −0.52 ± 2.55; *F* = 1.18, *p* = 0.328, *η* ^2^ = 0.022). SWLS showed no group difference (*p* = 0.519).

#### 3.4.6 Other measures

Imitation score did not show a significant group difference (*p* = 0.767).

### 3.5 Summary of findings

Table 5 summarizes the convergence of LMM and ANCOVA results across outcome domains. MMSE, MoCA visuospatial, gait speed, left contact time, right hip abduction strength, and bilateral leg segmental lean mass reached significance (*p* < 0.05) in both analyses. Cadence reached significance in ANCOVA (*p* = 0.045) with a trend in LMM (*p* = 0.092). Right single-leg stance time and SMI reached significance only in LMM (*p* = 0.026 and *p* = 0.020, respectively). WHOQOL social relationships reached significance only in ANCOVA (*p* = 0.046).

**TABLE 5:** Convergence of linear mixed model and ANCOVA results for variables reaching significance (*p* < 0.05) or trend († < 0.10) in at least one analysis.

Outcome	​	LMM	​	​	​	ANCOVA	​
	Direction	*β*	*p*	*β*_std	*F*	*p*	*η* ^2^
Cognitive function							
MMSE	Dance >	0.584	0.007**	0.32	7.55	0.022*	0.125
MoCA visuospatial	Dance >	0.266	0.023*	0.26	6.80	0.030*	0.114
MoCA abstraction	Dance >	0.154	0.063^†^	0.26	11.11	0.125	0.173
Gait parameters							
Gait speed (m/s)	Dance >	0.065	0.031*	0.17	3.07	0.018*	0.055
Contact time L (s)	Dance >	−0.018	0.005**	−0.32	5.22	0.033*	0.090
Cadence (steps/min)	Dance >	3.75	0.092^†^	0.18	1.61	0.045*	0.030
Balance and strength							
Hip abduction R (kg)	Dance >	1.22	0.016*	0.22	8.75	0.013*	0.142
One-leg stance R (s)	Dance >	10.34	0.026*	0.24	3.37	0.144	0.060
Grip strength R (kg)	Dance >	0.763	0.059†	0.15	3.80	0.092†	0.067
Knee extension L (kg)	Dance >	0.944	0.172	0.14	3.55	0.092†	0.063
Body composition	​	​	​	​	​	​	​
Seg. Lean LL (kg)	Control >	−0.108	<0.001**	−0.11	8.74	0.006**	0.142
Seg. Lean RL (kg)	Control >	−0.088	0.004**	−0.10	5.88	0.025*	0.100
SMI (kg/m^2^)	Control >	−0.064	0.020*	−0.10	1.92	0.192	0.035
Psychological wellbeing							
WHOQOL social	Dance >	0.367	0.120	0.19	3.86	0.046*	0.068

*β* = annual difference in change between dance and control groups (LMM). Positive values indicate greater improvement in the dance group for outcomes where higher scores are better; negative values for contact time and segmental lean mass indicate the dance group showed greater decrease. *β*_std = standardized beta (*β*/SD). *F* = *F*-statistic (ANCOVA). *η* ^2^ = partial eta-squared. Direction indicates which group showed more favorable change. Grey shading = control group showed more favorable change. ANCOVA *p*-values from permutation test (5,000 permutations). **p < 0.01, *p < 0.05, †*p* < 0.10.

Across both analyses, effects favoring the dance group were observed for cognitive function (MMSE, MoCA visuospatial), gait temporal parameters (speed, left contact time, cadence), and proximal lower-limb function (right hip abduction). In contrast, body composition outcomes (segmental lean mass, SMI) consistently showed greater decline in the dance group. Effect sizes ranged from *β*_std = 0.10–0.32 in LMM and *η* ^2^ = 0.055–0.142 in ANCOVA.

## 4 Discussion

This community-based, multi-region longitudinal study examined the effects of competitive hip-hop dance participation on cognitive function, gait, physical function, and body composition in community-dwelling older adults across three approximately annual assessments. Compared with active controls, dance participants showed better preservation of cognitive function, more favorable gait-related outcomes, and greater decline in muscle mass. These findings extend our previous short-term results (Miyazaki et al., 2022) and suggest that long-term dance participation may support cognitive and gait function, while being insufficient on its own to prevent age-related muscle loss.

### 4.1 Preservation of global cognitive function

One of the main findings of this study was better preservation of cognitive function in the dance group, particularly as reflected in MMSE scores. This finding is of interest because the MMSE is a widely used screening measure that covers multiple domains, including orientation, registration, attention, recall, and language (Folstein et al., 1975; Tombaugh and McIntyre, 1992). Unlike more domain-specific measures, change in MMSE may provide a broader indication of cognitive status, although it remains a screening instrument rather than a diagnostic test (Tombaugh and McIntyre, 1992). In the present study, the dance group showed a significantly more favorable trajectory than the control group (*β* = 0.58 points/year, *p* = 0.007), suggesting that continued dance participation may help preserve global cognitive function. This is consistent with meta-analytic evidence showing that dance improves global cognitive function in older adults (Hewston et al., 2021), and with recent reviews reporting cognitive and memory benefits in older adults with mild cognitive impairment (Tao et al., 2023).

This pattern extends our previous 4-week randomized controlled trial (Miyazaki et al., 2022), in which hip-hop dance improved MoCA and FAB scores but did not produce a detectable change in MMSE. The discrepancy may be attributable to the shorter intervention period and relatively high baseline MMSE scores in that earlier study. In the present three-wave longitudinal follow-up, the control group declined from T1 to T3 (28.3–27.2), whereas the dance group maintained MMSE performance over time. This suggests that the cognitive effects of dance may become more detectable on a global measure when participation is sustained over a longer period.

Mechanistically, dance may function as a concurrent cognitive-motor dual-task training; participants must continuously memorize, plan, and synchronize steps to music while performing physical exercise. This complex processing demand may support neuroplasticity and broader cognitive function over time (Hamacher et al., 2015; Kattenstroth et al., 2013). This interpretation is also broadly consistent with prospective observational evidence showing that participation in leisure activities, including dancing, is associated with lower risk of dementia in older adults (Verghese et al., 2003).

The significant effect observed in the MoCA visuospatial subscore (*β* = 0.27, *p* = 0.023) is also consistent with the demands of hip-hop dance. Participants were required to learn movement sequences, track their position relative to teammates, and adapt to spatial patterns in choreography. This interpretation is supported by previous evidence showing that dance training is particularly effective in improving visuospatial skills and inducing brain plasticity compared to repetitive physical exercise (Muinos and Ballesteros, 2021; Rehfeld et al., 2018). The competitive context of the FIDA GOLD CUP, in which teams performed coordinated routines before judges, may have intensified these visuospatial demands beyond those of typical recreational dance. MoCA abstraction also showed a trend in the LMM analysis (*β* = 0.15, *p* = 0.063), and the ANCOVA effect size was moderate (*F* = 11.11, *η* ^2^ = 0.173), although the permutation *p*-value did not reach significance (*p* = 0.125). Together, these findings suggest that the cognitive demands of choreography may extend beyond visuospatial processing alone, while the abstraction result should still be interpreted cautiously.

The real-world competitive setting may also have contributed to these cognitive effects. Participants practiced throughout the year with the shared goal of competition performance, resulting in sustained engagement from T1 through T3. This differs from the shorter duration of many research interventions. In addition, team practice required coordination, communication, and shared effort, all of which may have provided further cognitive stimulation. Indeed, social engagement and active participation in community-based group activities are well-known to contribute to cognitive improvement and lower the risk of cognitive decline (Penninkilampi et al., 2018). Several teams achieved notable competitive success during the study period, suggesting that the program successfully supported sustained engagement. Although the annual between-group difference in MMSE was numerically modest, the contrast between maintained performance in the dance group and decline in the control group may still indicate a meaningful difference over time.

### 4.2 Gait improvement without increased stride length

Another important finding of this study was that dance participants showed a more favorable pattern of gait-related change than controls, particularly reduced left contact time (*β* = −0.018 s/year, *p* = 0.005), while stride length remained unchanged. Cadence also showed a trend in the same direction (*β* = 3.75 steps/min/year, *p* = 0.092). This pattern suggests that the gait benefit of long-term hip-hop dance was driven more by temporal changes than by increased step length.

#### 4.2.1 Age-related decline in stride length

Stride length is known to decline with age and is a major contributor to reduced walking speed in older adults (Ko et al., 2010). Older adults may therefore rely on cadence as a compensatory strategy to maintain gait speed despite reduced step length (Jerome et al., 2015; Ko et al., 2010). This adaptation may also help avoid some of the balance demands (Lanza et al., 2022) and fear of falling (Makino et al., 2017) associated with longer steps. In addition, increasing stride length may be physically constrained in later life by reduced plantar-flexor force and limited hip extension, both of which restrict the ability to generate longer steps (Anderson and Madigan, 2014; Judge et al., 1996). Faster cadence has also been associated with less decline in walking speed over time in older adults (Jerome et al., 2015). In this context, the present findings suggest that cadence may play an important role in preserving gait performance in later life.

Age-related decline in walking speed becomes more apparent in later life, and longitudinal evidence suggests that the decline is greater in adults in their seventies than in those in their sixties (Sugiura et al., 1998). In the present study, the mean baseline age was 74.2 years, placing the cohort in an age range in which gait slowing may become more evident. As shown in Figure 3A, gait speed declined from T2 to T3 in the control group, whereas no comparable decline was observed in the dance group over the same period. This pattern is consistent with the possibility that continued dance participation may have helped attenuate age-related slowing of gait. Although the absolute change in gait speed was modest, preservation of walking speed in this later-life cohort may still be functionally relevant given the expected age-related slowing of gait.

#### 4.2.2 Rhythm as a possible mechanism

One interpretation is that hip-hop dance provided practice in moving in time with an external beat. Unlike conventional exercise, dance requires continuous synchronization between auditory rhythm and whole-body movement (Jin et al., 2019; Miura et al., 2011). Over time, this may have strengthened temporal control of stepping, allowing participants to maintain or improve gait performance without increasing stride length. This interpretation is consistent with previous work showing that rhythmic auditory cueing can improve spatiotemporal gait parameters in older adults (Ghai et al., 2018). In addition, studies of rhythmic training and sensorimotor synchronization suggest that movement to an external beat may strengthen temporal control of stepping over time (Dalla Bella et al., 2017; Thaut et al., 1996). In this sense, dance may function as a form of sensorimotor synchronization training through movement to music.

#### 4.2.3 Lateral stability and temporal gait adaptation

The side-specific pattern of findings may also help interpret the gait results. Right hip abduction strength improved significantly in the dance group (*β* = 1.22 kg/year, *p* = 0.016), whereas left hip abduction strength did not. In contrast, left contact time decreased significantly (*β* = −0.018 s/year, *p* = 0.005), whereas right contact time did not. Hip-hop dance includes frequent lateral stepping and weight shifts, which may place repeated demands on frontal-plane control. One possible interpretation is that improved right-sided lateral stability enhanced support during single-limb stance (Lanza et al., 2022; Martins et al., 2020), thereby contributing to shorter contact time on the contralateral side. Although this interpretation should be made cautiously, it is consistent with the role of the hip abductors in frontal-plane stability during walking (Arvin et al., 2015).

Several physical function measures showed non-significant trends favoring the control group, including TUG, heel-rise angle, and left hip flexion strength. These trends did not translate into more favorable gait outcomes and should be interpreted cautiously.

Taken together, these findings suggest that long-term hip-hop dance may support gait through a combination of temporal adaptation and improved lateral stability, rather than through increased stride length. However, this interpretation remains tentative because limb dominance, asymmetry, and frontal-plane kinematics were not assessed in the present study.

### 4.3 The muscle mass paradox

In contrast to the cognitive and gait findings, body composition outcomes favored the control group. Dance participants showed greater decline in skeletal muscle mass index (*β* = −0.064 kg/m^2^/year, *p* = 0.020) and segmental lean mass in both legs (left: *β* = −0.108 kg/year, *p* < 0.001; right: *β* = −0.088 kg/year, *p* = 0.004) than active controls. BMI did not differ between groups over time (*p* = 0.949). This dissociation suggests that the mechanisms supporting gait and cognition in the dance group were not necessarily accompanied by preservation of muscle mass.

One reason may be that hip-hop dance provided substantial rhythmic, coordinative, and aerobic stimulation, but did not provide the loading required to maintain muscle mass. Although participants moved regularly, the training stimulus may not have been sufficient in intensity or specificity to prevent age-related decline in lower-extremity muscle mass. This interpretation is consistent with the view that dance may support motor timing and coordination without necessarily functioning as resistance training (Hwang and Braun, 2015; Kattenstroth et al., 2013).

This issue may be particularly important in older adults because aging is associated with anabolic resistance, meaning that the muscle protein synthetic response to dietary protein intake is blunted (Breen and Phillips, 2011). Under such conditions, regular activity may not provide a sufficient anabolic stimulus to offset ongoing age-related muscle loss. Our previous pilot trial in frail nursing home residents also showed muscle mass decline despite participation in a rhythm-based group intervention (Miyazaki et al., 2020), whereas muscle mass was maintained in our later 4-week trial when amino acid supplementation was provided (Miyazaki et al., 2022). Together with the present findings, this pattern suggests that enjoyable movement-based interventions alone may not always be sufficient to preserve muscle mass in vulnerable older adults without nutritional support. However, because dietary intake, anabolic resistance, inflammatory status, and exercise intensity were not directly measured in the present study, these mechanisms should be interpreted cautiously.

Importantly, this does not diminish the value of dance itself. Rather, dance may be especially useful because it offers a form of physical activity that older adults can continue over time. In the present study, participants sustained team-based hip-hop dance across multiple assessments, suggesting that dance can provide an enjoyable, meaningful, and sustainable program even in later life (Predovan et al., 2019). Although the annual decline in SMI was numerically small, it may still be meaningful when considered cumulatively in older adults who are already at risk of sarcopenia. The practical implication is therefore not to replace dance, but to complement it with nutritional support or resistance training when preservation of muscle mass is also a goal.

### 4.4 Psychological wellbeing

Although no clear group × time interactions were observed in most psychological outcomes, the ANCOVA showed a significant group difference in the WHOQOL social relationships domain (dance *Δ* = +0.59, control *Δ* = −0.28; *F* = 3.86, *p* = 0.046, *η* ^2^ = 0.068). In contrast, depressive symptoms remained low and stable in both groups, and no clear group differences were observed for overall life satisfaction. This pattern is broadly consistent with meta-analytic evidence that dance movement interventions can support psychological health in older adults without dementia, although effects across specific domains remain variable (Podolski et al., 2023).

This may reflect the fact that the FIDA GOLD CUP provided more than physical activity alone. Participants practiced as teams throughout the year, prepared choreography together, and shared the goal of public performance in competition. These features may have fostered a sense of belonging, mutual support, and continued interpersonal engagement. In this respect, the social benefit observed here may reflect the long-term and collective nature of the program rather than dance alone.

This interpretation is also consistent with the contrast between the present study and our previous 4-week trial (Miyazaki et al., 2022), in which interventions were performed individually during the COVID-19 period and no clear improvement was observed in quality of life or depressive symptoms. Taken together, these findings suggest that the psychological value of dance for older adults may depend not only on movement itself, but also on sustained social participation.

### 4.5 Imitation ability

The gesture imitation task did not show a significant group × time interaction (LMM: *p* = 0.976; ANCOVA: *p* = 0.767), in contrast to our previous 4-week randomized controlled trial (Miyazaki et al., 2022), in which the dance group showed improved imitation scores relative to the walking group. A more likely explanation is the difference between meaningless gesture imitation and the more contextual motor learning involved in dance.

The task used in the present study assessed meaningless gestures, that is, novel hand configurations without clear functional or communicative meaning. Previous work suggests that meaningless gesture imitation declines with age, even after accounting for cognitive status (Baumard et al., 2020; Takasaki et al., 2024). It is therefore possible that long-term dance participation does not readily transfer to this type of context-free imitation task.

By contrast, learning and performing dance routines involves rhythm, music, social interaction, and goal-directed movement in a meaningful setting. These features differ substantially from copying arbitrary hand configurations in isolation. Indeed, previous research has shown that imitation performance is significantly better preserved when guided by a human trainer in a social context rather than a computer interface, as demonstrated even in Alzheimer’s patients (Bisio et al., 2016). Dance interventions in older adults have also been linked to motor-cognitive benefits under more complex task conditions, which may be more relevant to choreography learning than to decontextualized gesture imitation (Hamacher et al., 2015).

### 4.6 Strengths and limitations

This study has several strengths. First, it examined older adults participating in a real-world, community-based dance program with sustained engagement over multiple years, rather than in a short-term laboratory intervention. Second, the multi-site design increased ecological validity by including participants from several regions in Japan. Third, the study assessed a broad range of outcomes, including cognition, gait, lower-extremity function, body composition, psychological measures, and imitation ability, allowing a more comprehensive evaluation of the effects of long-term dance participation.

Several limitations should also be acknowledged. First, the study was not randomized, and participants in the dance group may have differed from controls in ways not fully captured by the measured covariates. Second, the sample was predominantly female, which may limit generalizability. Third, the control group was recruited from only two sites, raising the possibility of site-related confounding. Fourth, follow-up intervals varied across sites, although this was addressed analytically by modeling elapsed time from each participant’s actual assessment dates. Finally, although dance practice frequency and duration were recorded by questionnaire, exercise intensity, including metabolic equivalents and ratings of perceived exertion, was not directly measured in this cohort, limiting our ability to examine dose-response relationships. In addition, attrition over time, including the discontinuation of one team from follow-up competition participation, may also have influenced the longitudinal pattern of results.

We also note that multiple outcomes were examined, and several statistically significant findings were associated with modest or borderline *p* values. These results should therefore be interpreted as exploratory and hypothesis-generating rather than definitive, particularly for outcomes that were significant in only one analytic approach. Accordingly, we placed greater emphasis on findings that showed convergence across the linear mixed model and ANCOVA analyses.

### 4.7 Future directions

Future research should examine how the cognitive and gait benefits of dance can be maintained while also addressing age-related muscle loss. One important direction is the development of hybrid programs that combine dance with resistance training and nutritional support. Such programs may help preserve the motivational and social strengths of dance while adding the stimulus needed for muscle mass maintenance.

It will also be important to identify factors that support long-term participation. In the present study, attrition occurred over time, including discontinuation of follow-up at one site. Understanding barriers to continued engagement, and developing strategies such as flexible practice formats or additional support systems, may improve the feasibility of long-term community implementation.

Finally, further work is needed to clarify mechanisms. More detailed gait analysis, rhythm-related measures, and neurophysiological assessments may help explain how dance influences temporal gait control and cognitive function in older adults.

## 5 Conclusion

This multi-site longitudinal study showed that competitive hip-hop dance participation was associated with better preservation of cognitive function and more favorable gait-related change in community-dwelling older adults across three approximately annual assessments. Gait-related benefits were characterized by reduced contact time and a trend toward higher cadence, while stride length remained unchanged. This pattern suggests that long-term dance participation may support gait through temporal adaptation rather than increased step length.

At the same time, dance participants showed greater decline in skeletal muscle mass index and leg segmental lean mass than active controls. These findings indicate that the cognitive and gait benefits of dance do not necessarily extend to preservation of muscle mass.

Taken together, the results suggest that hip-hop dance may be a valuable community-based strategy for supporting cognitive and gait function in older adults, particularly when sustained participation is encouraged through meaningful group activity and shared performance goals. However, if prevention of age-related muscle loss is also a priority, dance may need to be combined with resistance training and nutritional support.

## Data Availability

The raw data supporting the conclusions of this article will be made available by the authors, without undue reservation.
